# Efficacy and Safety of Statins in MASLD and Other Chronic Liver Diseases

**DOI:** 10.3390/medsci14010084

**Published:** 2026-02-11

**Authors:** I. Commins, D. Clayton-Chubb, N. Janko, A. Majeed, W. Kemp, S. K. Roberts

**Affiliations:** 1 Department of Gastroenterology, Alfred Health, Melbourne, VIC 3004, Australia; 2 School of Translational Medicine, Monash University, Melbourne, VIC 3004, Australia; 3 Department of Gastroenterology, Eastern Health, Box Hill, VIC 3128, Australia

**Keywords:** statin, non-alcoholic fatty liver disease, MASLD, liver, dyslipidaemia, steatosis, elder

## Abstract

Metabolic dysfunction-associated steatotic liver disease (MASLD) is the most common liver disease worldwide, with an estimated global prevalence of 38% in adults. MASLD confers a significant increase in morbidity and mortality due to its association with cardiovascular disease and progressive liver disease, including cirrhosis and hepatocellular carcinoma. Current treatment paradigms for MASLD are centred around lifestyle modification and weight loss, with a need for pharmacotherapeutic options. Given the strong relationship between MASLD and cardiovascular disease, there is an interest in evaluating the efficacy and safety of cardiovascular medications such as statins in liver disease. Statins are the most commonly prescribed lipid-lowering medication in the world, with an established role in reducing cardiovascular morbidity and mortality. Statins are currently under-prescribed in the MASLD patient population, yet there is growing interest in determining whether statins could be utilised to treat MASLD itself. This comprehensive review aims to explore the evidence regarding the use of statin therapy for conventional, lipid-lowering indications in patients with MASLD and its potential benefits for the treatment of MASLD and its complications.

## 1. Introduction

### 1.1. Metabolic Dysfunction-Associated Steatotic Liver Disease

**Figure 1:**
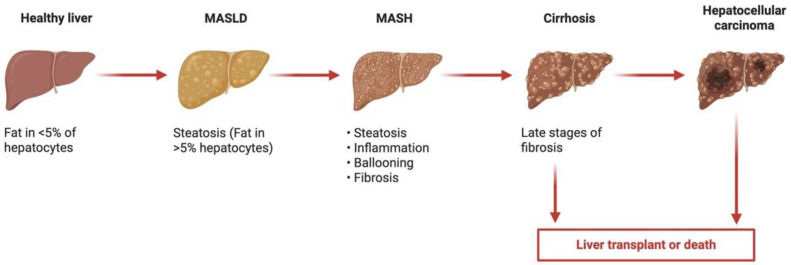
Metabolic dysfunction-associated steatotic liver disease (MASLD) spectrum and relevant histologic findings. Created in BioRender. Commins, I. (2026) https://BioRender.com/1u7njtn (accessed on 6 December 2025).

Metabolic dysfunction-associated steatotic liver disease (MASLD) is the most common liver disease worldwide, with an estimated global prevalence of 38% in adults, driven by increasing obesity and metabolic disorders [1]. MASLD confers a significant increase in morbidity and premature mortality via increased rates of cardiovascular disease (CVD) [2,3,4], as well as the potential progression of liver disease to cirrhosis and the development of hepatocellular carcinoma (Figure 1) [5]. Additionally, the world is transitioning to an older population [6,7], with recent data demonstrating that MASLD currently affects almost one-third of community-dwelling older Australians [8]. In older adults, MASLD is associated with frailty, social disadvantage, poor exercise tolerance, diabetes, and hypertension [8]. Current treatment paradigms for MASLD are centred around weight loss and optimisation of other medical comorbidities [9,10], with a vital need for pharmacotherapeutic agents to treat the condition itself. A detailed description of the pathophysiology of MASLD is beyond the scope of this paper. In brief, hepatic steatosis is defined as the presence of at least 5% of hepatocytes containing lipid vacuoles/triglycerides [11]. MASLD is identified in individuals with hepatic steatosis in the absence of so-called ‘secondary’ and genetic causes (e.g., prednisolone use, excess alcohol use, abetalipoproteinaemia), and in the presence of at least one cardiometabolic comorbidity [10]. Hepatic steatosis is often described as the result of a ‘multiple-hit’ phenomenon, where insulin resistance, adipokines, gut microbiotal products, nutritional intake, and genetic predisposition cause, in parallel and in tandem, the development of steatosis and potentially progressive fibrosis and cirrhosis [12]. This process is complex but involves dysregulated lipid metabolism, mitochondria-derived Reactive Oxygen Species (ROS), and inflammasome activation [12].

### 1.2. Statins

Japanese scientist Akira Endo discovered statins in the 1960s following his search for cholesterol-lowering agents [13]. Subsequent Nobel-prize winning work by Michael Brown and Joseph Goldstein showed that statins inhibit HMG-CoA reductase and consequently reduce low-density lipoprotein (LDL) [14]. It is well established that the primary mechanism of action of statins is the competitive blocking the active site of HMG-CoA reductase, the first and rate-limiting enzyme in the mevalonate pathway [15]. Within the liver, this leads to an inhibition of hepatic cholesterol biosynthesis, leading to the upregulation of hepatic LDL receptors, thus increasing the clearance of LDL-cholesterol (LDL-C) from the bloodstream [16]. This leads to a reduction in circulating LDL-C levels by 22–55% [17]. However, in addition to reducing LDL-C, statins have other non-lipid related, pleiotropic effects, thought to be due to inhibition of the synthesis of isoprenoid intermediates of the mevalonate pathway [17]. These include improvements in endothelial function; stabilisation of atherosclerotic plaques; anti-inflammatory, immunomodulatory, and anti-thrombotic effects; positive effects on bone metabolism; and a reduction in the risk of dementia [15]. Given the inextricable link between MASLD and metabolic dysfunction, there is good reason to believe that many of the pleiotropic benefits of statins may have clinical benefits in both MASLD and the progressive form of the disease, metabolic dysfunction-associated steatohepatitis (MASH), through reducing hepatic steatogenesis, as well as protecting against fibrosis and hepatocellular carcinoma (HCC) [18].

Statins are the most widely used lipid-lowering drug in the world. In the United States, nearly 30% of adults over 40 years old are taking a statin [19]. Furthermore, in 2011, the British Heart Foundation reported that one million statin prescriptions were provided each week in England [20]. The choice of statin in clinical practice is based on several factors, including potency, subtype (hydrophilic vs. lipophilic), and other concurrent medication use. The most potent of the statins are rosuvastatin and atorvastatin. These are used as first-line, high-intensity, LDL-C lowering therapy [21]. Statins can be further divided into lipophilic (simvastatin, fluvastatin, and atorvastatin) and hydrophilic (pravastatin and rosuvastatin) statins. The lipophilic group can more easily enter cells, whereas hydrophilic statins have greater hepatoselectivity [22]. Although the benefits of statins in the primary and secondary prevention of cardiovascular disease is well established, the superiority of hydrophilic vs. lipophilic statins is not [22]. The class difference has the greatest clinical significance with respect to adverse effects, with statin-associated muscle symptoms (SAMs) more commonly associated with lipophilic statin therapy [23].

Given the strong relationship between MASLD and cardiovascular disease, there is a significant focus on the safety and efficacy of cardiovascular medicines in liver disease. Statins are effective for both primary and secondary prevention of atherosclerotic cardiovascular disease (ASCVD) [24] via not only LDL-C reduction but also potentially through other important pleiotropic effects [25]. Historically, statins have been under-prescribed in the MASLD patient population, given concerns around hepatotoxicity [26,27]. However, the safety of statin use in liver disease, and particularly MASLD, is well established [28,29,30,31]. Recent research has demonstrated that statin use for primary prevention in MASLD patients has increased over time, but that guideline-based use remains low [32].

This review will aim to explore the evidence demonstrating benefits of statin therapy, not only for the conventional lipid-lowering indications in MASLD, but also for the potential benefits of using statin therapy for the treatment MASLD. In exploring the effects of statin therapy on the complications of chronic liver disease (e.g., portal hypertension and hepatocellular carcinoma), other aetiologies of liver disease, in addition to MASLD, will also be explored.

## 2. Materials and Methods

In order to formulate a review of the current literature on the efficacy and safety of statins in liver disease, in particular in MASLD and in cirrhosis of any aetiology, the electronic databases, MEDLINE and Cochrane CENTRAL, were searched from their inception until September 2025. The search was performed using multiple keywords in combination, including HMG-CoA Reductase Inhibitors, Statins, Fatty Liver, Non-Alcoholic Fatty Liver, Hypolipidemic Agents, Liver Disease, Liver Cirrhosis, Portal Hypertension, Metabolic Dysfunction-Associated Steatotic Liver Disease, Steatotic Liver Disease, and Metabolic and Alcohol Associated Liver Disease. Given the global nomenclature change from non-alcoholic fatty liver disease (NAFLD) to MASLD [33], our search strategy used both terms to capture the largest relevant literature possible. This approach is supported by work showing almost complete clinical concordance between definitions [34,35], including in older persons [8]. Studies from any country were assessed; however, studies were limited to those in the English language only. Pre-clinical and non-human studies were included if they were determined to be useful to expand on the relevant concepts, specifically if they reported on the in vitro effects of statins on various aspects of hepatic function. Additional material was identified through the References section of relevant papers and personal knowledge of the literature. Only published papers were included.

## 3. Statins and Metabolic Dysfunction-Associated Steatotic Liver Disease (MASLD)

**Figure 2:**
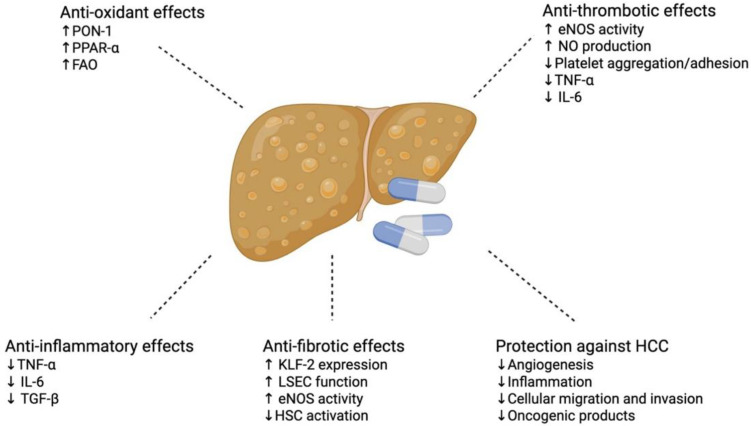
Pleiotropic effects of statins in MASLD/MASH. PON-1 = paraoxanase-1, PPAR-α = peroxisome proliferator-activated receptor alpha, FAO = fatty acid oxidation, eNOS = endothelial nitric oxide synthase, NO = nitric oxide, TNF-α = tumour necrosis factor alpha, IL-6 = interleukin 6, TGF-β = transforming grown factor beta, KLF-2 = Kruppel-like factor 2, LSEC = liver sinusoidal endothelial cell, HSC = hepatic stellate cell, and HCC = hepatocellular carcinoma. Created in BioRender. Commins, I. (2026) https://BioRender.com/1u7njtn (accessed on 6 December 2025).

As previously described, the pleiotropic (potent anti-inflammatory, anti-fibrotic, anti-oxidative, and anti-thrombotic) effects of statins have plausible clinical benefits in both MASLD and MASH, such as reducing hepatic steatosis and fibrosis and protecting against hepatocellular carcinoma [36] (Table 1, Table 2 and Table 3) (Figure 2).

### 3.1. Association Between Statin Use and Hepatic Steatosis

The association between statins and MASLD has been well studied in both observational and several randomised controlled trials, and the results are generally positive, with many studies suggesting that statins might reduce the risk of developing MASLD through a reduction in hepatic steatosis [30,37,38,39,40,41,42,43]. A Cochrane meta-analysis on the effect of statins on MASLD and MASH based on two small trials (with a high risk of bias) found that statins improve both serum aminotransferase levels and sonographic hepatic steatosis; however, they did not demonstrate improvements in liver histology or liver-related mortality and morbidity [44]. One randomised controlled trial showed a reduction in the odds of hepatic steatosis by 71% after 4 years of a combination of atorvastatin 20 mg/daily, vitamin C, and vitamin E in participants with radiographically diagnosed MASLD, although it is impossible to ascertain how much of the treatment effect is due to the statin alone [45]. Although statins have been shown to have an association with a reduction in hepatic steatosis, people without dyslipidaemia (and not on a statin therapy) seem to have the lowest risk of MASLD, indicating that statins do not entirely ameliorate the risk for MASLD [18]. Importantly, other studies have also demonstrated that a higher dose and longer duration of statin has a significant effect on the development and progression of MASLD [37,39,46,47]. In vitro studies on 3D cultured human liver organoids found that higher concentrations of statins significantly inhibited the number of lipid droplets [18]. However, the question of dose and duration of the statin on the effects of MASLD progression remains essentially unanswered and warrants further research. Despite the majority of evidence pointing towards a benefit on hepatic steatosis with statin use, there have been two randomised controlled trials (RCTs) that did not show an improvement in hepatic steatosis with statin use: one of these trials was small, with a high risk of bias [48], and the other used a lower-potency statin, pitavastatin [49].

### 3.2. Association Between Statin Use and Metabolic Dysfunction-Associated Steatohepatitis

Simple hepatic steatosis, with or without inflammation, is thought to have a generally benign course; however, the progressive form of MASLD, MASH, can further progress to fibrosis and subsequently cirrhosis, resulting in liver failure and hepatocellular carcinoma [50]. Statins have been shown to be inversely associated with MASH, indicating that statins may be hepatoprotective [18,46,51,52]. One randomised controlled trial looked at the effect of rosuvastatin in combination with N-acetyl-cysteine (NAC) for the treatment of MASH, compared to treatment with only Vitamin E, and found a reduction in the mean value of hepatic steatosis by 16.49% (*p* = 0.017), as well as a reduction in the mean fibrosis value of 19.5% (*p* = 0.001) [53]. The potential benefits of statins for the treatment of MASH have also been shown in patients with diabetes, a key risk factor for progressive MASLD [54], highlighting the potential protective effects of statins in high-risk patients, as well as their potential to alter the natural history of the disease [55].

### 3.3. Association Between Statin Use and Hepatic Fibrosis

Experimental MASH models have shown that statins can inhibit the paracrine signalling between hepatocytes and hepatic stellate cells (HSCs), resulting in the de-activation of HSCs and halting fibrogenesis [56,57]. In addition to the promising findings of the in vitro studies, the effect of statins on fibrosis in observational trials has also been favourable. Multiple studies have shown that statins are negatively associated with hepatic fibrosis, often in a dose-dependent manner [38,46,47,52,53,55,58,59]. The mechanism behind this is thought to be the potent anti-inflammatory, anti-oxidant, and anti-thrombotic effects of statins, which confers a certain level of protection against steatohepatitis and fibrosis [60]. One randomised controlled trial looking at patients with chronic hepatitis C infection and concurrent MASLD demonstrated that the addition of rosuvastatin to their hepatitis C therapy reduced hepatic steatosis and fibrosis [61]. Some studies have failed to show improvements in fibrosis or histological inflammation, though the majority of these were small and thus, underpowered [18,28,29,30,45,48,62]. The positive results regarding statin use and fibrosis should be interpreted with caution, given that most trials in this area have been observational in nature. However, given the clear safety of statins in MASLD and the promising results of statins in improving fibrosis to date, this warrants further research.

### 3.4. Associations Between Statin Use and Non-Liver Related Outcomes

MASLD and MASH are independent risk factors of cardiovascular disease [4,63], and current evidence suggests that statins lead to a significant reduction in cardiovascular morbidity and mortality in patients with MASLD and concurrent dyslipidaemia [28,29,30,64,65]. One review highlighted that statin treatment halves the mortality and morbidity associated with ASCVD in patients with MASLD/MASH, and additionally, that statins reduce cardiovascular events by two-thirds in patients with MASLD/MASH compared to those not on a statin [64]. As such, current clinical practice guidelines recommend the use of statins (with or without other lipid-lowering agents) for the treatment of dyslipidaemia in MASLD [9,10,66]. Furthermore, guidelines also include recommendations of cardiovascular optimisation with a statin prior to liver transplant in patients with MASLD, including with cautious, case-by-case usage in decompensated liver disease [9,10]. A Cochrane review examining two randomised controlled trials looking at the effects of statins on MASLD/MASH did not find a mortality or morbidity benefit in trial participants on a statin. However both trials included small numbers of participants and high levels of bias; therefore, more robust randomised controlled trial data is required [44].

**Table 1:** Randomised controlled trials of statins for the treatment of MASLD/MASH. MASLD = metabolic dysfunction-associated steatotic liver disease, MASH = metabolic dysfunction-associated steatohepatitis, LDL-C = low density lipoprotein cholesterol, LFTs = liver function tests, CT = computed tomography, ^1^H-MRS = proton magnetic resonance spectroscopy, NAS = NAFLD activity score, FIB-4 = Fibrosis 4 index for liver fibrosis score, and IHCL = intrahepatocellular lipid.

Authors, Year	Patient Population, (Number of Participants)	Relevant Study Methods	Primary Study Findings
Athyros et al., 2006 [41]	Adult, non-diabetic patients with metabolic syndrome and ultrasonographic evidence of MASLD at baseline (*n* = 186).	Patients with dyslipidaemia were randomised to atorvastatin (20 mg/day) or fenofibrate (200 mg/day), or both for 54 weeks.	A total of 67% of patients on atorvastatin, 42% on fenofibrate, and 70% on combination no longer had biochemical plus US evidence of MASLD (*p* < 0.05 for all).
Nelson et al., 2009 [48]	Adult patients with biopsy-proven MASH (*n* = 16).	Patients were randomised to either simvastatin 40 mg daily or placebo, for 12 months.	A 26% reduction in LDL-C in simvastatin group. No statistically significant improvement in liver function tests, hepatic steatosis, necroinflammatory activity, or stage of fibrosis within or between groups.
Athyros et al., 2010 [28]	Adult patients with coronary heart disease (aged < 75 years, serum LDL-C > 2.6 mml/L and triglycerides < 4.5 mml/L) and deranged LFTs likely due to MASLD (*n* = 437).	Patients were randomised to statin therapy (mainly atorvastatin 24 mg per day) or usual care; 227 patients received a statin; 210 patients did not. Follow up over 3 years.	There was an improvement in liver function tests in patients who received statin therapy over 3-year follow up. Statin treatment reduced the risk for cardiovascular events by 68% in patients with abnormal LFTs (*p* < 0.0001).
Foster et al., 2011 [45]	Adult patients with CT-proven MASLD (*n* = 80).	Patients were randomised to daily atorvastatin 20 mg, vitamin C 1 g, and vitamin E 1000 IU vs. placebo, with median follow up of 3.6 years.	Atorvastatin 20 mg in combination with vitamins C and E is effective in reducing the odds of having hepatic steatosis by 71% after 4 years of therapy.
Malaguarnera et al., 2011 [61]	Adult patients with chronic hepatitis C and concurrent MASLD (*n* = 65).	Patients were randomised to either leukocyte interferon alpha (3 MIU 3 times/week) plus ribavirin (1200 mg/day) or interferon alpha plus ribavirin, at the same doses, and rosuvastatin (5 mg/day) for 12 months.	The addition of rosuvastatin to interferon and ribavirin significantly reduced viraemia, hepatic steatosis, and hepatic fibrosis.
Braun et al., 2018 [49]	Overweight, insulin-resistant, adult males (*n* = 50).	Patients were randomised to either pitavastatin 4 mg daily or placebo for 12 weeks.	Pitavastatin use showed no change in liver fat fraction as measured by ^1^H-MRS.
Sfikas et al., 2021 [43]	Adult patients with MASLD/MASH (*n* = 604).	Patients were randomised into four groups: diet/exercise, atorvastatin, rosuvastatin, or pitavastatin for 12 months.	After 12 months, the diet/exercise group showed no significant change in NAS/FIB-4. Atorvastatin, rosuvastatin, and pitavastatin produce a beneficial and safe effect in NAFLD/NASH patients, as recorded by the improvement in the NAS and FIB-4 scores (*p* < 0.001 for all).
Wang et al., 2024 [42]	Adult, non-diabetic patients with the metabolic syndrome and intrahepatocellular lipid levels > 10% as determined by ^1^H-MRS (*n* = 32).	Patients were randomised to either rosuvastatin 10 mg daily or placebo for 12 months.	Rosuvastatin resulted in a significant absolute (△IHCL: 7.61 ± 4.51 vs. 1.54 ± 5.33, p = 0.002) and relative (△IHCL%: −42.28 ± 24.90% vs. −8.91 ± 31.93%, *p* = 0.003) reduction in IHCL compared to the results for the control.

**Table 2:** Systematic reviews/meta-analyses of statins for the treatment of MASLD/MASH. RCTs = randomised controlled trials, MASLD = metabolic dysfunction-associated steatotic liver disease, NAFLD = non-alcoholic fatty liver disease, and NAS = NAFLD activity score.

Authors, Year	Relevant Study Methods	Primary Study Findings
Fatima et al., 2021 [52]	Observational studies and RCTs that assessed the efficacy of statins for the treatment of MASLD and its development. A total of 14 studies were included.	The authors found that statins may significantly reduce the risk of developing NAFLD (OR:0.69, 95% CI [0.57,0.84]; *p* = 0.0002). Statin use significantly improved liver function tests. Steatosis grade, NAS, and necro-inflammatory stage all underwent significant reduction.
Abdallah et al., 2022 [31]	A 21 placebo-controlled RCT including 1900 patients (304 receiving statins, 520 other lipid lowering therapies, and 61 combinations) treated for a mean 26 weeks (IQR 17.5–52).	People taking a statin showed lipid profile improvement without any worsening of ALT, AST, total bilirubin, or alkaline phosphatase at the end of the study; NAS did not improve with statin use. There was no change in fibrosis stage with statin use.

**Table 3:** Other randomised trials of statins for the treatment of MASLD/MASH. MASLD = metabolic dysfunction-associated steatotic liver disease, LDL-C = low density lipoprotein cholesterol, MRI-PDFF = magnetic resonance imaging-derived proton density fat fraction, MASH = metabolic dysfunction-associated steatohepatitis, FAST score = [eˆ(–1·65 + 1·07 × In(LSM) + 2·66 × 10^−8^ × CAP^3^ − 63.3 × AST^−1^)]/[1 + eˆ(–1·65 + 1·07 × In(LSM) + 2·66 × 10^−8^ × CAP^3^ − 63.3 × AST^−1^)] [67], and NAC = N-acetyl cysteine.

Authors, Year	Patient Population, (*Number of Participants*)	Relevant Study Methods	Primary Study Findings
Athyros et al., 2011 [65]	Adult patients with sonographic evidence of MASLD (*n* = 326).	Patients were treated with atorvastatin over 42 months in a dose-titrating manner with the intention to attain the LDL-C target <130 mg/dL in half the patients and <100 mg/dL in the other half.	Lipid levels and liver function tests normalised, liver ultrasonographic findings associated with NAFLD resolved, and no cardiovascular events occurred in patients that attained the LDL-C target of <110 mg/dL. Three CVD events occurred in the group that did not attain the LDL-C target (*p* = 0.024).
Mitsiou et al., 2018 [39]	Adult patients with well controlled blood pressure (*n* = 40).	Patients were randomised to low-dose (5 mg/day) or high-dose (20–40 mg/day) rosuvastatin for 6 months.	Both groups had normalisation of liver enzymes, but the group on high dose rosuvastatin had a greater improvement in liver steatosis as measured by hepatic ultrasound (*p* = 0.01)
Cho et al., 2022 [40]	Adult patients with ultrasound-proven MASLD (*n* = 70).	Patients were randomised to receive either ezetimibe 10 mg plus rosuvastatin 5 mg daily or rosuvastatin 5 mg daily for up to 24 weeks.	Reduction in hepatic steatosis as assessed by MRI-PDFF in combination group (18.1 to 12.3%, *p* < 0.001) and monotherapy group (15.0 to 12.4%, *p* = 0.003). No effect on fibrosis seen in either group.
Zakaria et al., 2025 [53]	Adult patients with MASH as diagnosed by the FAST score (*n* = 90).	Patients were randomised to vitamin E 400 IU twice daily for 6 months or NAC (gemacystein) 1200 mg twice daily with rosuvastatin 20 mg daily.	Reduction in mean value of hepatic steatosis by 16.49% in the group treated with rosuvastatin and NAC (*p* = 0.017). Reduction in mean fibrosis value of 19.5% (*p* = 0.001) in patients treated with rosuvastatin and NAC, in addition to improvement in metabolic parameters and health-related quality of life scores.

## 4. Statins for the Treatment of Chronic Liver Disease

**Figure 3:**
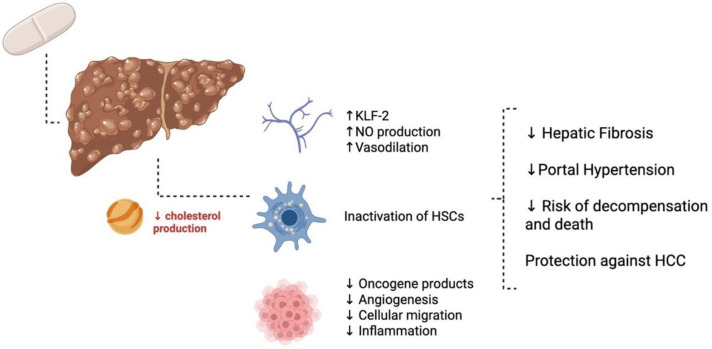
Proposed clinical benefits of statins in advanced chronic liver disease. KLF-2 = Kruppel-like factor 2, NO = nitric oxide, HSC = hepatic stellate cell, and HCC = hepatocellular carcinoma. Created in BioRender. Commins, I. (2026) https://BioRender.com/1u7njtn (accessed on 6 December 2025).

The non-lipid lowering, pleiotropic effects of statins show promise for the treatment of cirrhosis. Current pre-clinical evidence is suggestive that statins have the ability to decrease oxidative stress and inflammation, improve endothelial function and hepatic vascular tone, decrease stellate cell turnover, and provide protection from lipopolysaccharide-mediated damage and ischaemic reperfusion injury (Figure 3) [68].

### 4.1. Pharmacokinetics of Statins in Chronic Liver Disease

Given that the primary mechanism of action of all statins is to inhibit HMG-CoA reductase, an important part of preventing of endogenous cholesterol production in the liver, all statins are relatively hepatoselective. The degree of hepatoselectivity is determined by the solubility profile of the statin [69]. Lipophilic statins passively diffuse through the hepatocyte cell membrane, leading to efficient first-pass uptake, whereas hydrophilic statins undergo carrier-mediated uptake, thus leading to greater hepatoselectivity [70]. This is clinically significant, as hydrophilic statins have less muscle penetration, and therefore, a lower prevalence of statin-associated muscle symptoms (discussed in further detail below) [15]. The main route of elimination for most statins, after metabolism by the liver, is via the bile [69]. Therefore, hepatic dysfunction is a risk factor for adverse events such as statin induced myopathy (discussed below), and all statin manufacturers recommend caution in patients with a history of liver disease [71]. Pravastatin and rosuvastatin undergo elimination by both the kidney and the liver. Nevertheless, pravastatin’s pharmacokinetics are still altered in the setting of hepatic dysfunction; however, rosuvastatin’s are not [69]. Clinical data on the pharmacokinetics of the various statins in liver disease is somewhat limited due to there being only a small number of studies of statins in patients with chronic liver disease. A recent systematic review of the pharmacokinetics of statins in cirrhosis reported data for most of the statins currently on the market, excepting lovastatin and simvastatin [72]. The authors found that the largest change in area under the curve (AUC) and maximum plasma concentration (Cmax) was found in Child–Pugh B patients taking atorvastatin, indicating an 11-fold and 16-fold increase, respectively [72]. The smallest change in AUC was reported in Child–Pugh A patients taking rosuvastatin 10 mg daily [73], and the smallest change in Cmax was in Child–Pugh A patients after a single dose of pitavastatin 2 mg [74]. In general, the authors found that higher AUC and Cmax results were seen in Child–Pugh B compared to Child–Pugh A patients [75]. Additionally, there is an increase in both AUC and Cmax of different statins in patients with Child–Pugh A and B cirrhosis compared to the results for non-cirrhotic patients, with the degree of increase dependent on the severity of liver impairment [72]. At present, there is no data to suggest if any dose-adjusting method is superior for dosing statins in cirrhosis [72].

### 4.2. Hepatic Fibrosis

Statins may have a beneficial role in halting the progression of chronic liver disease. The mechanism is largely thought to be statin’s ability to downregulate the expression of profibrotic cytokines, which prevent the activation of hepatic stellate cells and subsequent fibrinogenesis [76,77,78,79]. In addition, statins can also lead to the upregulation of Kruppel-like factor 2 (KLF-2) expression that causes vasodilation and improves liver microcirculation [76,77,78,79]. A recent systematic review and meta-analysis examining the effects of statins on the progression of hepatic fibrosis, of which all patients had either hepatitis B- or C-related chronic liver disease, showed that statins halted the progression of fibrosis, the development of cirrhosis, and hepatic decompensation events [80]. In addition, in patients with chronic HCV infection, a decrease in mortality was also observed in those patients taking a statin. This review did not find any evidence that statins were harmful in patients with chronic liver disease, noting that patients with decompensated cirrhosis were not included in the studies. Additionally, a recent cohort study of 16,501 patients with chronic liver disease, encompassing several aetiologies of liver disease including hepatitis C, alcohol related liver disease, and MASLD, demonstrated that patients who were on a statin were more likely to transition from high to intermediate or low FIB-4 score (a non-invasive, composite laboratory and age-based score to assess the risk of fibrosis [81]) groups and less likely to remain in the high group than were non-users [82].

### 4.3. Portal Hypertension

Portal hypertension is the main driver of hepatic decompensation events in patients with cirrhosis, leading to increased morbidity and mortality [83]. Although non-selective beta blockers remain part of the standard of care for patients with portal hypertension, up to 45% of patients fail to achieve sufficient portal pressure reduction [84]. There is burgeoning interest in the use of statins as an additional therapeutic option to improve portal hypertension. In cirrhosis, an inadequate release of nitric oxide contributes to increased hepatic resistance and portal pressure, which in turn enhances the post prandial increase in portal pressure [85]. There are thought to be a few mechanisms through which statins improve portal hypertension. Transcription factor KLF-2 protects endothelial cells through the induction of vasoprotective genes. In cirrhosis, KLF-2 is overexpressed early during the progression of the disease. However, it does not slow down the development of vascular dysfunction [57]. Simvastatin has been shown to activate the KLF-2–nitric oxide pathway. Upregulation of this pathway induces a profound improvement in portal hypertension, endothelial dysfunction, and liver fibrosis. The beneficial effects of KLF-2 are due to the inactivation and apoptosis of hepatic stellate cells, as well as a reduction in hepatic oxidative stress and improvement in endothelial function [57]. Several studies have demonstrated that simvastatin use in patients with cirrhosis and portal hypertension leads to an overall decrease in hepatic venous pressure gradient (HVPG), with haemodynamic effects greater in those with more severe portal hypertension (Table 4) [85,86,87,88], with one study also demonstrating a survival benefit in those with Child–Pugh A and Child–Pugh B cirrhosis [89]. A recent systematic review and meta-analysis found that statins were associated with a significant HVPG reduction, along with a higher haemodynamic response when compared with placebo. However, there was no significant difference seen in regards to variceal bleeding, ascites, or mortality [84]. In studies comparing statin plus non-selective beta blocker (NSBB) to NSBB alone, the addition of statins to NSBB therapy further reduced HVPG [88,90]. As such, statins may be a useful adjunct to NSBBs; however, further data are required to assess the long-term clinical benefits.

**Table 4:** Randomised controlled trials of statins for the treatment of portal hypertension. ETOH = alcohol, HCV = hepatitis C virus, HBV = hepatitis B virus, HVPG = hepatic venous pressure gradient, AIH = autoimmune hepatitis, BD = twice daily, and PBC = primary biliary cholangitis.

Authors, Year	Patient Population (*Number of Participants*)	Aetiology of Liver Disease	Intervention/Control	Primary Study Findings
Abraldes et al., 2009 [87]	Adult patients with cirrhosis and severe portal hypertension (HVPG ≥ 12 mmHg) (*n* = 55).	ETOH (*n* = 23), HCV (*n* = 27), HBV (*n* = 2), other (*n* = 3).	Simvastatin 20 mg/day for 1 month (increased to 40 mg/day at day 15)/placebo.	Simvastatin significantly decreased HVPG by 8.3%. HVPG decreases were observed in patients receiving concurrent beta blockers (−11.0%, *p =* 0.033) and in those who were not (−5.9%, *p* = 0.013).
Pollo-Flores et al., 2015 [86]	Adult patients with cirrhosis and portal hypertension (*n* = 34).	HCV (58%), ETOH (17%), HBV (17%), AIH (8%).	Simvastatin 40 mg/day for 2 months/placebo.	A total of 55% of patients in the simvastatin group presented a clinically relevant decrease in the HVPG (at least 20% from baseline or to ≤ 12 mmHg); no decrease was seen in the placebo group (*p* = 0.036). The haemodynamic effect appeared more evident in those with severe portal hypertension.
Bishnu et al., 2018 [88]	Adult patients with cirrhosis and clinically significant portal hypertension (*n* = 23).	ETOH (*n* = 10), HBV (*n* = 1), NASH (*n*= 1), AIH (*n* = 1), Wilson’s disease (*n* = 1), cryptogenic (*n* = 9).	Atorvastatin 20 mg daily with propranolol in incremental doses/incremental dose propranolol for 30 days.	Decrease in HVPG in atorvastatin group vs. control group 2.58 ± 1.88 vs. 4.81 ± 2.82 mmHg (*p* =0.041). No significant difference in clinical outcomes (variceal bleed, endoscopic variceal ligation sessions, hepatic encephalopathy, requirement of therapeutic paracentesis, spontaneous bacterial peritonitis, and death).
Vijayaraghavan et al., 2020 [91]	Adult patients with cirrhosis and portal hypertension HVPG > 12 mmHg and small or large oesophageal varices (*n* = 220).	ETOH (*n* = 83), NASH (*n* = 90), HBV (*n* = 17), HCV (*n* = 19).	Carvedilol (escalated to maximum dose) plus simvastatin (20 mg daily, escalated to 40 mg daily) for 3 months/carvedilol only for 3 months.	The degree of mean HVPG reduction (17.3% and 17.8%, respectively, *p* = 0.98) and hemodynamic response was not different between the statin/carvedilol and carvedilol only groups. Three (3.7%) patients on simvastatin developed transient transaminitis and elevated creatine phosphokinase (all three patients had Child–Pugh C cirrhosis) and improved with drug withdrawal.
Kronborg et al., 2023 [92]	Adult patients with cirrhosis and portal hypertension (*n* = 78).	ETOH (*n* = 66), MASLD (*n* = 4), AIH (*n* = 1), PBC (*n* = 3).	Atorvastatin 10–20 mg/day for 6 months/placebo.	The study confirmed the safety of atorvastatin but did not demonstrate a reduction in mortality, the risk of liver-related complications, or the HVPG.
Alvarado-Tapias et al., 2024 [90]	Adult patients with cirrhosis and high-risk varices (*n* = 82).	ETOH (*n* = 34), HCV (*n* = 28), ETOH + HCV (*n* = 7), MASLD (*n* = 7), Others (*n* = 6).	Carvedilol (escalated to maximum dose) + simvastatin 20 mg daily/xarvedilol + placebo for 4 weeks.	HVPG significantly decreased in both groups: carvedilol + simvastatin (18.6 ± 4 to 15.7 ± 4 mm Hg, *p* < 0.001); carvedilol + placebo (18.9 ± 3 to 16.9 ± 3 mm Hg, *p* < 0.001). The decrease was significantly larger with carvedilol + simvastatin (2.97 ± 2.5 vs. 2.05 ± 1.6 mm Hg, *p* = 0.031).

### 4.4. Risk of Decompensation and Mortality

Decompensation events in patients with cirrhosis represent a significant change in both clinical status and prognosis [83]. One recent randomised controlled trial looking at simvastatin 20 mg/d plus rifaximin 1200 mg/day vs. placebo in patients with either Child–Pugh B or C cirrhosis did not find any significant difference in outcomes between the two groups in terms of acute-on-chronic liver failure, transplant, complications, cirrhosis, or death [93]. Additionally, there have been several observational studies looking at the effects of statins on decompensation and mortality in cirrhosis of various aetiologies. The results of these studies are largely positive, with statin use found to be associated with a lower risk of decompensation [94,95] and death [94,96,97,98,99]. In addition, statins have also been associated with a lower risk of hospitalisation for infections [100] and acute-on-chronic liver failure [101]. Although the results from these observational studies appear positive, the current data are significantly limited, in part due to the retrospective and observational nature of the studies. In addition, statin dose and duration were not accurately measured, and the population included well patients with compensated cirrhosis, therefore perhaps over-estimating the benefits of statins in cirrhosis [68]. The cohorts studied were largely people with viral hepatitis; therefore, generalizability to other aetiologies of cirrhosis may not be possible. However, this certainly remains an area of interest which warrants prospective studies to further elucidate the effect of statins on decompensation and mortality in cirrhosis.

### 4.5. Hepatocellular Carcinoma

Statins have been shown to decrease the incidence and recurrence of a variety of types of malignancies. Studies have suggested that this occurs through the inhibition of cholesterol synthesis but also due to statins’ effects on malignant signalling pathways and on oncogene products with effects on inflammation, cellular migration, invasion, and angiogenesis [102,103,104,105]

More than 20 retrospective analyses have shown an association between statins and a lower incidence of HCC across various aetiologies of liver disease. The majority of studies have been performed on patients with viral hepatitis in Asian populations [68]. However, there have also been promising results in the MASLD population. Some studies have shown that patients with MASLD have a lower incidence of HCC if taking statin therapy [106,107]. One large cohort study demonstrated that statin use was associated with a significant reduction (25%) in the risk of HCC (as well as decompensation and mortality) in patients with diabetes and concurrent MASLD cirrhosis [108]. In patients with HCC who underwent resection or transplantation, HCC recurrence was seen less frequently in patients taking statins [109,110]. However, there was no overall survival benefit in this population. Overall, the current evidence suggests that statin therapy is associated with a reduced incidence of HCC and may have some benefit following diagnosis of HCC, but prospective randomised data is needed.

### 4.6. Safety in Cirrhosis

Despite the current literature pointing to the safety of statin use in advanced chronic liver disease, statins are often under-prescribed due to safety concerns [68]. One study found that only 23% of patients with coronary artery disease and cirrhosis undergoing liver transplant evaluation were on a statin [111]. It is well established that the pharmacokinetics of statins are altered cirrhosis, particularly in decompensated disease, due to impaired synthetic function [112]. However, despite this, multiple studies have shown a low incidence of hepatic injury or hepatic decompensation in patients with cirrhosis and statin exposure [68,113,114]. It must be noted, however, that the inclusion of patients with advanced or decompensated disease in most studies is limited. Statins have been safely used in patients with advanced chronic liver disease, even in decompensated cirrhosis, but the risk of statin-induced adverse events might be higher in this population (discussed in more detail below) [28].

## 5. Statin Choice in Chronic Liver Disease

The choice of statin therapy, including the dosing regimen, must take into consideration both safety and efficacy. Statins differ in both solubility (hydrophilic vs. lipophilic) and metabolism (i.e., whether metabolised by the cytochrome P450 system or not) [68]. It is worth highlighting that with respect to efficacy in atherosclerotic cardiovascular disease (ASCVD), there is no data to date that shows a difference in outcomes between hydrophilic and lipophilic statins [68]. Most of the randomised trials looking at the use of statins for the treatment of MASLD/MASH have studied either atorvastatin or rosuvastatin, with largely positive results (Table 1). The trial using pitavastatin did not show the same benefits as those seen with the other two more potent statins (Table 1). As aforementioned, rosuvastatin has also been shown to improve hepatic fibrosis in patients with both MASLD and chronic hepatitis C infection [61]. In patients with other aetiologies of advanced chronic liver disease, data is limited regarding statin choice. One study found that atorvastatin and fluvastatin were associated with the most effective anti-fibrotic effects in a cohort of patients with non-cirrhotic hepatitis C [115].

There is currently minimal data related to statin choice in people with cirrhosis, as clinical studies have predominantly been limited to simvastatin, atorvastatin, and pravastatin [72]. Although no specific statin has shown a consistent benefit over other statins with respect to all-cause mortality or progression of cirrhosis, lipophilic statins (simvastatin and atorvastatin) were more strongly associated with reduced incidence of HCC and mortality in a nationwide viral hepatitis cohort [116]. This finding has been subsequently confirmed in other studies [117,118]. Additionally, as mentioned earlier, several studies have shown that simvastatin leads to a significant decrease in HVPG in in patients with cirrhosis and portal hypertension [85,86,87,88].

## 6. Statins and Safety

The prevalence of statin intolerance is difficult to ascertain, particularly with respect to muscle symptoms [119]. Current observational and clinical trial data suggests that intolerance occurs in as many as 10–30% of patients [120,121]. With respect to statin use in liver disease, current clinical practice guidelines recommend that statins should be use in adults with chronic liver disease, including in those with compensated cirrhosis. They should be used in adults according to cardiovascular risk guidelines to reduce cardiovascular events [10]. The safety and efficacy of individual statins is summarized in Table 5.

### 6.1. Statin-Associated Muscle Symptoms (SAMSs):

SAMSs are the most common adverse events associated with statin use, comprising over 70% of all reported adverse events in the general population [122]. SAMSs can be divided into four groups:

1. Rhabdomyolysis characterised by elevated creatinine kinase (CK) concentrations (>100-fold the upper limit of normal), myoglobinuria, and renal impairment.
2. Myalgia or mildly elevated CK (<5× ULN).
3. Self-limited toxic statin myopathy (CK levels between 10 and 100× ULN).
4. Myositis or immune-mediated necrotizing myopathy with HMG-CoA reductase antibodies and CK levels between 10 and 100× ULN [15].

SAMSs often affect the large proximal muscles (often of the lower extremities) in a symmetric fashion. The risk is highest in the first year of therapy, with dosage increases and in the setting of polypharmacy [68]. Prevalence differs between classes, the highest risk involving lipophilic statins (simvastatin, atorvastatin, and lovastatin). Hydrophilic statins such as pravastatin have less muscle penetration and therefore, are less associated with SAMSs [15]. Other risk factors include high statin dose, older age, frailty, female sex, low BMI, and concurrent ethanol (ETOH) use [68]. There are also genetic risk factors for SAMSs [123]. Caution should be taken when statins are used in combination with fibrates due to a higher risk of statin-induced myopathy [9]. The prevalence of SAMSs in people with cirrhosis is comparable to that of the general population; however, more data are needed, particularly in cases of advanced disease [68]. In one large RCT evaluating statin use (simvastatin 40 mg daily), 19% of patients with decompensated cirrhosis experienced rhabdomyolysis [124]. Simvastatin is extensively metabolized by the liver, with a first-pass effect, and is highly protein bound [89]. Therefore, overall, caution is suggested for use in advanced cirrhosis (Child–Pugh > 9), given the limited data for this patient population, as well as avoidance of high-dose simvastatin [68].

### 6.2. Liver Function Test Derangement

Statins have been associated with a dose-dependent effect on transaminases; however, this effect is often mild (<3–5× ULN), transient, asymptomatic, and does not require discontinuation of therapy [125,126,127,128]. It appears to be a class effect, with the mechanism likely linked to changes in the hepatocyte membrane lipid composition [15,129]. Furthermore, transient ALT elevation is higher in lipophilic statin- rather than hydrophilic statin-treated patients [130]. One meta-analysis of over 50,000 people demonstrated no difference in LFTs between statin and placebo groups after 12 weeks of therapy [131]. Severe, idiosyncratic drug-induced liver injury (DILI) is rare [132], and progression to liver fibrosis has not been observed [30]. In addition, statin-induced autoimmune hepatitis is rare but described, and can be treated with immunosuppression [133]. Another meta-analysis of around 50,000 patients treated with statins confirmed the safety of patients with MASLD and elevated LFTs. The authors also found that patients with hyperlipidaemia and MASLD have fluctuations in transaminases, regardless of whether they are treated with a statin or not [134]. Current clinical practice guidelines suggest that patients with chronic liver disease and/or mild elevations in serum transaminases should be considered eligible for satin use. Furthermore, many patients with mild elevations in liver enzymes likely have concurrent MASLD and would benefit the most from the initiation and continuation of lipid-lowering therapy [68]. Interestingly, there has been good data to suggest that patients with MASLD taking a statin actually show an improvement in their serum aminotransferase levels, likely reflecting the improvement in hepatic steatosis associated with statin use [28,44,135,136]. Given the fact that mild ALT elevations have not been shown to be associated with true hepatotoxicity or changes in liver function, routine monitoring of ALT during statin therapy is no longer recommended in clinical practice guidelines [25].

### 6.3. New-Onset Diabetes Mellitus

New-onset diabetes mellitus (NODM) has been associated with statin use and is predominantly seen in older patients with pre-existing metabolic comorbidities receiving high-dose statin therapy [23,137]. It appears to be a class effect, with NODM developing with both hydrophilic and lipophilic statin use [138]. The mechanism by which statins cause NODM is complex and remains poorly understood. Several pathophysiologic mechanisms have been postulated; however the end result is that of reduced insulin secretion and reduced insulin sensitivity [138]. Data from meta-analyses has shown that statin therapy is associated with a 9% increased risk of developing NODM, and that high-dose statin, compared to moderate-dose statin, is associated with a higher incidence of NODM [139,140]. In addition, the higher potency statins (atorvastatin, rosuvastatin, and simvastatin) appear to have a stronger association with NODM [141]. However, despite this increased risk of NODM, the data still demonstrates an overwhelming benefit in terms of the reduction of cardiovascular events, and therefore, the benefits of intensive statin therapy significantly outweigh the risk of NODM. This benefit has also been confirmed in a primary prevention setting with a lower-risk population, where the overall beneficial effect of statin therapy on vascular events was greater (almost 2.5 fold) than the hazard of developing NODM [142]. This potential adverse event caused by statins needs to be carefully considered in the context of patients with MASLD, given their predisposition to having higher insulin resistance compared to that of the general population. For example, it may be prudent to monitor Hba1c more closely in patients with MASLD without diabetes who are taking a statin. Further prospective research is required to assess the risk of patients with MASLD developing NODM while taking a statin.

### 6.4. Cognitive Impairment

Statins have been linked to short-term, reversible cognitive impairment since their inception. A phase 1 clinical trial of atorvastatin demonstrated that escalating doses showed a dose-dependent adverse reaction of mild, transient mental confusion, together with restlessness and euphoria [143]. However, these effects were not reported to in the phase 2 and 3 trials to the degree that they could be recognised as side effects [144]. Subsequently though, post-marketing data has revealed an ill-defined memory loss which is reversible upon discontinuation of the statin, with most reports coming from people over the age of 50 [145]. In 2012, the Food and Drug Administration (FDA) announced a label change for all statins, informing prescribers and patients that cognitive side effects are a risk when taking statins [144]. It is currently unknown whether cognitive impairment occurs at a similar frequency in patients with liver disease; however, some research has shown that hepatic encephalopathy, a condition seen in progressive liver disease, is observed less frequently in the setting of statin use [101].

### 6.5. Other Adverse Drug Reactions

Other reported statin-mediated adverse events include cataracts, urogenital side effects, gynecomastia, mild proteinuria, haemorrhagic stroke, gastrointestinal effects, and reproductive effects. Most of these have been postulated to occur as a result of reduced production of the products of the mevalonate pathway [15].

### 6.6. Drug–Drug Interactions

There are several important drug interactions with statins that may increase the risk of adverse effects, particularly myopathy and rhabdomyolysis [25]. Most available statins (except pravastatin, rosuvastatin, and pitavastatin) undergo major hepatic metabolism via the cytochrome P450 (CYP) system. CYP3A4 isoenzymes are the most implicated in the metabolism of statins (others being CYP2C8, CYP2C9, CYP2C19, and CYP2D6). Therefore, other pharmacological substrates of the CYPs may interfere with statin metabolism and vice versa, and statin therapy may interfere with the metabolism of other implicated medications [25,146]. In addition to their implications in the CYP system, statin inhibition or induction of P-glycoprotein (P-gp), an intracellular tissue-specific transport system, may also result in drug interactions [147]. Drug interactions have been reported between statins and P-gp substrates, including digoxin, diltiazem, and verapamil [147]. Importantly, there is a significant overlap between agents that are both CYP3A4 and P-gp substrates or inhibitors. Research has demonstrated that atorvastatin, lovastatin, and simvastatin inhibited P-gp mediated transport, and co-administration with these statins and other P-gp substrates or inhibitors may result in increased bioavailability of these statins and thus, an increased risk of adverse effects [147].

**Table 5:** Safety and efficacy of individual statins. LDL-C = Low-density lipoprotein cholesterol.

Statin	Dosage Intensity [148]	LDL-C Reduction % [148]	Major Clearance Pathway [69]	Safety Considerations
Rosuvastatin	High Intensity: 20 to 40 mg	≥50%	Both hepatic and renal	Benign, low-grade proteinuria at high doses, often transient [149].
	Moderate Intensity: 5 to 10 mg	30%–<50%		
Atorvastatin	High Intensity: 40 to 80 mg	≥50%	Mainly hepatic	Potential for increased rates of adverse effects (dose-related myopathy and rhabdomyolysis) with CYP3A4 inhibitors [146].
	Moderate Intensity: 10 to 20 mg	30%–<50%		
Simvastatin	Moderate Intensity: 20 to 40 mg	30%–<50%	Both hepatic and renal	Potential for increased rates of adverse effects (dose-related myopathy and rhabdomyolysis) with CYP3A4 inhibitors [146].
	Low Intensity: 10 mg	<30%		
Lovastatin	Moderate Intensity: 40 to 80 mg	30%–<50%	Mainly hepatic	Potential for increased rates of adverse effects (dose-related myopathy and rhabdomyolysis) with CYP3A4 inhibitors [146], requires dose adjustment in CKD [150].
	Low Intensity: 20 mg	<30%		
Pravastatin	Moderate Intensity: 40 to 80 mg	30%–<50%	Both hepatic and renal	Low interaction burden [151].
	Low Intensity: 10 to 20 mg	<30%		
Pitavastatin	Moderate Intensity: 1 to 4 mg	30%–<50%	Mainly hepatic	Low interaction burden [151]; requires dose adjustment in CKD [150].
Fluvastatin	Moderate Intensity: 40 mg 2×/day; XL 80 mg	30%–<50%	Mainly hepatic	Low interaction burden [151].
	Low Intensity: 20 to 40 mg	<30%		

## 7. Safety and Efficacy of Statins Between the Sexes

There is well-established evidence examining the efficacy and safety of statins between the sexes. Meta-analysis data looking at the efficacy of statins in both sexes found that the benefits of statins in reducing MACE and all-cause mortality occurs equally between men and women [152,153]. The difference between the sexes is largely in the safety and tolerability of statins. Women report higher rates of SAMSs compared to men; this effect appears to increase with age and is dose-dependent [154,155]. Although relatively few discontinue statin therapy, women are more likely to discontinue statin therapy compared to men [156]. Additionally, there have been higher reported rates of new-onset diabetes mellitus associated with statin use in women compared to in men [157]. Furthermore, women are more prone to the nocebo and drucebo effects of statins [155]. Specifically, in patients with MASLD, there has also been evidence highlighting that statins are under-prescribed in women compared to men, [158]. The reasons for the differences in statin use between sexes includes that women are offered statin therapy less commonly than men, and women tend to discontinue statin therapy more frequently than men [156,158,159]. Greater clinician awareness of gender-specific side effects would hopefully lead to improved clinician sensitivity during prescribing and thus, an improved adherence to statins among women. This is of paramount importance in the MASLD population, given the association with atherosclerotic cardiovascular disease and major adverse cardiovascular events [3].

## 8. Statins in Older Adults

The world is rapidly transitioning towards an older population, with almost twice as many people surviving beyond 80 years now compared to the numbers noted 50 years ago [6,7]. Furthermore, >80% of people who die from cardiovascular disease are >65 years of age [25]. However, despite this, the use of statin therapy declines with increasing age, likely reflecting a difference in both prescription and adherence when compared to younger cohorts [160]. The effect of statins on older people has been relatively understudied, with few patients >75 years of age included in statin trials [161,162,163]. While the benefits of statins in the treatment of ASCVD is clear across all age groups, in people without ASCVD, the benefits are not as clear in older adults (Table 6). As such, there are currently no strong prescribing guidelines for older adults without ASCVD, a limitation that is currently being addressed by the STAtin Therapy for Reducing Events in the Elderly (STAREE) trial [164]. With respect to the adverse effects of statins in older adults, these remain of particular concern due to increased comorbidities such as chronic kidney disease, polypharmacy, and altered pharmacokinetics and pharmacodynamics that occur with ageing [25]. However, as with efficacy, the extent of statin adverse events in older adults has not been well studied [145]. Drug interactions are a concern, as discussed above, and as such, statins are recommended to be commenced at low dose if there is significant renal impairment or potential for drug interactions [25]. With respect to hepatic adverse events, a recent retrospective cohort study looking at 167,112 adverse events due to statins, of which there were 7779 reported cases of DILI, the authors found that DILI cases predominantly occurred in older patients (≥65 years), with 45.43% of cases occurring in that age group [165]. Additional case report data has supported the finding that statin-induced DILI predominantly affects older adults [129,166]. However, there is currently not enough evidence to suggest a dose reduction of statins in older adults to prevent DILI. Nonetheless, given the ageing population and clear benefits of statins in younger adults, more prospective data is warranted to study both the efficacy and safety of statins in older adults, particularly in those with MASLD.

**Table 6:** Efficacy and safety of statins in certain subpopulations. MASLD = metabolic dysfunction-associated steatotic liver disease, MASH = metabolic dysfunction-associated steatohepatitis, LDL-C = low-density lipoprotein cholesterol, MACE = major adverse cardiovascular events, and ASCVD = atherosclerotic cardiovascular disease.

Sub-Population	Efficacy	Safety Considerations
Pre-diabetes	Statins lower rates of MACE in people with impaired fasting glucose and known ASCVD [167].	Modest increase in new-onset diabetes (~9–12% relative risk), greatest with high-intensity therapy [139,140]. Higher new-onset diabetes risk with more potent statin therapy (atorvastatin and rosuvastatin); lower risk with pravastatin and pitavastatin [139]. Benefits in treatment of ASCVD with statin still outweigh the risks of developing diabetes [168].
Type 2 diabetes	Statins reduce all-cause mortality, vascular mortality, and MACE in people with diabetes, with and without ASCVD [169,170].	Statins may cause a small increase in Hba1c; this is seen more commonly with atorvastatin use [168]. Low-dose statin therapy is not recommended in people with diabetes, given their increased risk of ASCVD; rather, moderate–high intensity therapy should be used, depending on the exact indication [170].
Older adults (≥70 years)	Clear benefits in elderly people with known ASCVD; however, unclear role in primary prevention [164].	Adverse events in older people not well studied [145]; drug interactions are a concern in polypharmacy [25]; DILI occurs more commonly in older people [165]. Statins should be commenced at a low dose if there is significant renal impairment or potential for drug interactions [25].
MASLD/MASH	Statins reduce cardiovascular morbidity and mortality in patients with MASLD and concurrent dyslipidaemia [28,29,30,64,65].	The safety of statin use in liver disease, and particularly MASLD, is well established, including in compensated cirrhosis [28,29,30,31]. Liver function tests often improve in patients with MASLD taking a statin [28,44,135,136].

## 9. Statins in MASLD—Unanswered Questions

Pre-clinical and clinical trials to date have shown that statins may be beneficial in preventing MASLD, MASH, and hepatic fibrosis. There are also promising results with respect to the potential benefits advanced chronic liver disease and its complications, as well as reductions in hepatocellular carcinoma. However, large, prospective trials remain scarce, particularly those involving robust histological endpoints to examine the effects of statins on MASH and hepatic fibrosis. Furthermore, although many studies have revealed positive results, some of the available evidence remains contradictory or inconclusive. Further studies are required to examine the hepatoprotective effects of statins in patients with MASLD who do not have a conventional indication for statin therapy, i.e., in primary prevention.

## 10. Conclusions

As highlighted by this review, statins are one of the most prescribed medications worldwide and are highly effective in the treatment and prevention of ASCVD. Despite the well-established safety of statins in MASLD, statins remain under-prescribed in patients with MASLD, a condition commonly associated with the same co-morbidities as ASCVD. Most importantly are the cardiovascular benefits, with data showing that statin treatment halves the mortality and morbidity associated with ASCVD in patients with MASLD/MASH. Additionally, statins reduce cardiovascular events by two-thirds in patients with MASLD/MASH compared to in those not on a statin. There is growing evidence that statins may in fact improve hepatic steatosis and fibrosis, carry benefits in cirrhosis with portal hypertension, and reduce the risk of hepatocellular carcinoma. Therefore, statins should be more widely considered in patients with MASLD not only to reduce the progression and complications of disease such as MASH and fibrosis but also to reduce cardiovascular events, mortality, and morbidity. Increased awareness amongst physicians, particularly in the primary care setting, is needed to improve the uptake of statin therapy in MASLD patients. Further prospective studies are warranted to examine whether statins are hepatoprotective in MASLD patients without a conventional indication for statin therapy.

## Data Availability

The original contributions presented in this study are included in the article. Further inquiries can be directed to the corresponding author.

## Abbreviations

The following abbreviations are used in this manuscript:

MASLD	metabolic dysfunction-associated steatotic liver disease
CVD	cardiovascular disease
ROS	reactive oxygen species
LDL	low-density lipoprotein
HCC	hepatocellular carcinoma
LDL-C	how-density lipoprotein cholesterol
SAMs	statin-associated muscle symptoms
ASCVD	atherosclerotic cardiovascular disease
MASH	metabolic dysfunction-associated steatohepatitis
NAC	N-acetylcysteine
HSC	hepatic stellate cell
RCT	randomised controlled trial
AUC	area under the curve
Cmax	maximum plasma concentration
KLF-2	Kruppel-like factor 2
HVPG	hepatic venous pressure gradient
NSBB	non-selective beta blocker
CK	creatinine kinase
ETOH	ethanol
ULN	upper limit of normal
NODM	new-onset diabetes mellitus
FDA	Food and Drug Administration
CYP	cytochrome P450
P-gp	P-glycoprotein
STAREE	STAtin Therapy for Reducing Events in the Elderly
DILI	drug-induced liver injury
CT	computed tomography
LFTs	liver function tests
^1^H-MRS	proton magnetic resonance spectroscopy
NAS	NAFLD activity score
FIB-4	Fibrosis 4 Index for Liver Fibrosis
IHCL	intrahepatocellular lipid
NAFLD	non-alcoholic fatty liver disease
MRI-PDFF	magnetic resonance imaging-derived proton density fat fraction
HCV	hepatitis C virus
HBV	hepatitis B virus
AIH	autoimmune hepatitis
BD	twice daily
PBC	primary biliary cholangitis
PON-1	paraoxanase-1, PPAR
PPAR-α	peroxisome proliferator-activated receptor alpha
FAO	fatty acid oxidation
eNOS	endothelial nitric oxide synthase
NO	nitric oxide
TNF-α	tumour necrosis factor alpha
IL-6	interleukin 6
TGF-β	transforming grown factor beta
LSEC	liver sinusoidal endothelial cell
